# “You First Have to Check Out the Doctors”: Transition Expectations and Experiences of Survivors After Pediatric Cancer

**DOI:** 10.1002/cam4.70455

**Published:** 2024-12-10

**Authors:** Ekaterina Aleshchenko, Thorsten Langer, Gabriele Calaminus, Juliane Glogner, Henrike Haugke, Pietro Trocchi, Enno Swart, Katja Baust

**Affiliations:** ^1^ Institute of Social Medicine and Health Systems Research, Faculty of Medicine Otto von Guericke University Magdeburg Germany; ^2^ University Hospital of Schleswig‐Holstein, Campus Luebeck Luebeck Germany; ^3^ Department of Pediatric Hematology and Oncology University Hospital Bonn Bonn Germany

**Keywords:** cancer survivorship, pediatric cancer, stereotype priming model, theory of planned behavior, transition from pediatric to adult healthcare

## Abstract

**Purpose:**

The shift from child‐centered to adult‐focused healthcare presents social and psychological challenges for adolescents and young adults with chronic conditions, which can affect their participation in follow‐up care. This study aims to investigate the factors that influence patient‐driven motivations for adhering to follow‐up recommendations, while also exploring the barriers and supports that impact the transition process for pediatric cancer survivors.

**Methods:**

We developed interview guidelines grounded in the Theory of Planned Behavior (TPB) and the stereotype priming model (SPM). We conducted 10 episodic narrative interviews with pediatric cancer survivors during their transition phase. The interview transcripts were analyzed through qualitative thematic content analysis, followed by quantitative analysis using multiple regression models. This research is a part of the broader VersKiK study, which aims to propose enhancements to the organization of (long‐term) follow‐up care after cancer in childhood and adolescence in Germany.

**Results:**

In this study, pediatric cancer survivors transitioning to adult healthcare showed varied motivations for follow‐up care. Positive attitudes, driven by understanding the benefits, improved engagement, while anxiety and fear of losing familiar care created barriers. Family and peer support played a key role in encouraging adherence. Perceived control over healthcare management, supported by organizational help, was crucial, but logistical and financial challenges often undermined this control.

**Conclusions:**

Several motives underlying pediatric cancer survivors' behavior significantly influence their transition experiences. Enhancing positive attitudes, strengthening social support, and improving perceived control through targeted interventions may support smoother transitions to adult healthcare facilities.

**Trial Registration:** Registered at German Clinical Trial Register (IDs: DRKS00025960 and DRKS00026092)

## 1 Background

The transition from child‐centered to adult‐focused healthcare for adolescents and young adults with chronic conditions is a planned process that introduces new social and psychological challenges, affecting their participation in (long‐term) follow‐up care [1]. Survivors often need additional motivation and environmental support to cope with these challenges. Without such support, young adults may show reduced adherence to treatment recommendations, interruptions in medical follow‐up, development of medical complications, or deterioration in overall health [2].

While these challenges may apply to all medically vulnerable young adults, it is essential to focus specifically on pediatric cancer survivors—a growing group, with approximately 2100 new cancer diagnoses annually, and a survival rate exceeding 80% in Germany [3]. Despite being cured of cancer, pediatric cancer survivors are at risk for late effects, including long‐term morbidities, resulting from the original cancer or its treatment (e.g., [4, 5]). Consequently, effective disease prevention and (long‐term) follow‐up care management for this group of survivors rely on their transition to and continued involvement in the adult healthcare system [6]. Despite the significant medical relevance, a US study has revealed that over 50% of pediatric cancer survivors are not receiving organized cancer‐related follow‐up care [7]. Another US study involving 11,000 childhood cancer survivors found that less than half of those at high risk participated in follow‐up monitoring for secondary cancers (12.6%) and cardiac issues (41.4%) [8]. Additionally, Alchin et al. found that pediatric cancer survivors exhibited low adherence rates and had poor recall of their healthcare recommendations, with only 45% and 28% remembering the advice at 1 and 6 months postsurvivorship consultation, respectively [9]. This is due to various factors, including issues related to both the survivors and the healthcare system [7]. One possible reason might be that pediatric survivors are frequently asymptomatic as they are to be transited into adult healthcare facilities, which may reduce their motivation to stay engaged with (long‐term) follow‐up care and other health‐promoting behaviors [10, 11]. Additionally, pediatric cancer survivors might experience psychological symptoms such as depression, anxiety, and posttraumatic stress, which can impede their engagement to follow‐up care and make the transition a process with challenging emotional aspects [12, 13]. Lastly, only a small group of adult healthcare providers, primarily based in specialized multidisciplinary centers associated with university hospitals, is knowledgeable about the complex health issues encountered by pediatric cancer survivors. This pushes survivors and their informal caregivers to manage follow‐up care within the adult healthcare system mostly by themselves, thus requiring strong self‐advocacy skills [6, 14].

The Theory of Planned Behavior (TPB) is a commonly used framework for understanding health behaviors, focusing on internal motivations through three interconnected aspects: attitude, subjective norm, and perceived behavioral control [15]. Attitude involves individuals' assessments of the positive and negative outcomes linked to a behavior. Subjective norm relates to the perceived social desirability and norms of “significant others” to perform the behavior, such as the expectation from parents that they should attend follow‐up care. Perceived behavioral control concerns an individual's ability to carry out the behavior, encompassing both the actual capability and the confidence to do so.

Additionally, health behavior can be unconsciously influenced by broader social and environmental stimuli, known as “primes” according to Bargh et al. [16, 17]. Internal motivations are crucial to attend (long‐term) follow‐up care and impact the intention to adhere to guideline‐based behavioral recommendations. Internal motivations may play an important role in attending (long‐term) follow‐up care and could influence the intention‐to‐follow guideline‐based behavioral recommendations.

Recently, several university clinics in Germany have set up multidisciplinary long‐term follow‐up clinics [18], providing specialized, risk‐adapted monitoring for pediatric cancer survivors in line with current guidelines [19]. These clinics also offer the opportunity for transitional appointments, where both a pediatric oncologist and an internist are present to thoroughly discuss the transition from pediatric to adult healthcare systems. However, their numbers remain limited, and there is a need for better dissemination of information about their availability.

To study the transition experiences of pediatric cancer survivors, we are aiming to: (1) investigate potential factors affecting their motivations to adhere to follow‐up recommendations and attend recommended examinations in (long‐term) follow‐up care and (2) collect their insights about barriers and facilitators of the transition process.

## 2 Methods

This article presents our analyses of interview transcripts in a subgroup of transition survivors collected alongside a broader interview study [20]. This study is a part of the larger VersKiK project to research the long‐term effects of childhood and adolescent cancer, adherence to particular (long‐term) follow‐up guidelines, and actual (long‐term) follow‐up needs of survivors and their informal caregivers. The overall study design of VersKiK and a detailed description of the design of this study are presented elsewhere [20, 21].

### 2.1 Design

To investigate the motivations and needs of pediatric cancer survivors, we created episodic narrative interview guidelines in German [22]. At the beginning of the interview, pediatric cancer survivors or their caregivers were asked an open question about their “survivorship story,” encouraging them to share spontaneous narratives about their experiences. To identify “primes,” or unconscious stimuli related to health behavior, they also provided associations with the terms “cancer,” “follow‐up,” and “survival” [17]. The interview guideline had a modular structure, with each block containing an introductory question, a five‐dimensional Likert‐scale question (intensity or agreement scale [17]), and a subquestion for more specific insights. Each block focused on elements of the TPB [15, 23] or the topics of “information needs” and “transition.” We decided to ground the interviews in two interconnected theories to address the limitations of a purely TPB‐based approach by considering subconscious primers that influence behavior and underlie most action intentions [24]. The comprehensive process of developing the interview guideline is described elsewhere [25].

### 2.2 Recruitment

We employed convenience sampling [26] to recruit participants, reaching out through specialized channels such as the German magazine for childhood cancer patients, survivors, and parents (WIR), social media platforms of the Children Cancer Foundation (https://www.kinderkrebsstiftung.de/), and collaborating hospitals. We aimed to gather a diverse group of participants aged 17–45 years old (adolescents and young adults), varying in diagnoses and duration of (long‐term) follow‐up. Eligibility criteria included survivors of any treatment type and cancer diagnosis, regardless of cancer stage or grade, currently being in the transition phase. The sole exclusion criterion was an inadequate cognitive function that might hinder participation in the interview. The final sample size was determined by thematic saturation, which occurs when further data do not provide new insights, as themes and comments from participants start to repeat [27]. Additionally, the small sample size can be justified by the interview structure, which allowed for in‐depth discussions of the transition experiences in each case [28]. Lastly, the final sample size aligns with previous research, as shown by Vasileiou et al. [29].

### 2.3 Data Collection

EA and JG carried out 10 interviews across multiple locations: the University Hospital Schleswig‐Holstein and the University Hospital Bonn, both of which have specialized multidisciplinary long‐term follow‐up care, and the University Hospital Magdeburg, which lacks such facilities. These interviews took place between September 2022 and March 2023, mostly with survivors recruited directly before or after their transition appointment. Written consent was obtained from all participants.

### 2.4 Analyses

We analyzed interview transcripts twofold—defined general themes characterizing the needs of pediatric cancer survivors in the transition (qualitative) and their motivations to adhere to follow‐up recommendations (qualitative and quantitative).

To generate topics from interview transcripts, we followed Hsieh and Shannon's five‐step approach to data analysis [30]. This involved transforming text into narrative form, identifying units of analysis and themes, establishing coding rules, implementing the coding system across all narrative data, refining it as needed, and finally, validating and selecting the ultimate dataset. Themes were initially defined by EA and then reviewed by KB. Through thematic coding, we systematically classified significant concepts and recurring themes within the narratives. Codes were assigned based on these recognized themes and further subdivided as required to accurately capture the essence of the messages.

To identify and describe factors affecting (long‐term) follow‐up adherence, we analyzed the interview transcripts both qualitatively and quantitatively based on TPB dimensions. We analyzed themes quantitatively based on the dimensions of TPB using thematic content analyses [31]. To explore the influence of different TPB factors and consider the effects of unconscious primes, we have taken the following steps. To investigate unconscious primes underlying health behavior, participants were asked to provide associations to the terms “cancer,” “follow‐up,” and “survival” at the beginning of the interview. Two researchers (EA and PT) independently evaluated these associations and categorized them as positive, negative, or neutral, assigning corresponding points 1.5, 0.5, or 1 (variable: association factor). Agreement between unconscious stimuli and the extent of consent was determined by comparing Likert‐scaled answers with the association factor (variable: agreement). Agreement was present if the mean Likert score was > 2 and the mean value of the association factors was > 1 or if the mean Likert score was < 2 and the mean association factor was < 1, therefore showing in the same direction. There is no agreement if the mean Likert score was > 2 and the mean association factor was < 1 or if the mean Likert score was < 2 and the mean association factor was > 1. Second, regression models were then employed to identify which dimensions most significantly influenced the intention of adult survivors and informal caregivers to attend follow‐up appointments, assessing the relative strength of predictors using standardized beta coefficients and comparing them across dimensions. However, due to small sample size, the regression analyses had exploratory meaning and aimed to support and validate our qualitative findings. This methodological approach is considered a starting point for subsequent quantitative studies on determinants of adherence to long‐term follow‐up.

**TABLE 3:** Results from multiple regression models for predictors of intention‐to‐attend follow‐up by dimension of TBH: Standardized beta coefficients.

	Social norm	Perceived control	Attitude	Intention
Age at diagnosis	0.47	−0.36	−0.51	−0.21
Time since diagnosis	0.13	−0.16	0.30	−0.58
Diagnosis	−0.42	−0.45	−0.41	−0.00
Relapse	−0.01	0.23	−0.06	−0.00
F. Up care visit	0.34	−0.50	0.09	0.01
Association factor (with “primes”)	0.98	−0.98	−1.50	0.22
Agreement between stimuli and content	−0.57	1.51	1.63	0.36

## 3 Results

The general characteristics of the transition subgroup of interviewees are presented in Table 1.

**TABLE 1:** General characteristics transition subgroup of interviewees.

	Year of birth	Diagnosis	Year since diagnoses	Age at diagnosis	Relapse
1	2000	Osteosarcoma	13.2	9	N
2	2006	Osteosarcoma	4.3	12	N
3	2006	HL	1.5	15	N
4	2005	Osteosarcoma	2.6	15	N
5	2002	Ewing's sarcoma	4.0	16	N
6	2003	ALL	6.0	13	N
7	2004	Nephroblastoma	10.4	8	N
8	2003	Neuroblastoma	5.6	14	Y
9	1978	HL	17.3	27	N
10	2004	CML	5.7	13	N

Abbreviations: ALL, acute lymphoblastic leukemia; CML, chronic myeloid leukemia; HL, Hodgkin's lymphoma; N, no; Y, yes.

### 3.1 Needs in Transition

We identified general survivorship themes relevant to the survivors during the transition, as well as specific themes related to the transition itself. Summarized themes, subthemes, and referring dimensions of TPB are presented in Table 2.

**TABLE 2:** Interview themes.

Themes	Subthemes	Dimensions of TBH
Relevance of returning to everyday life	Challenges in reintegration into social life and impact of physical limitations	**Attitudes:** Positive and negative beliefs about reintegration
		**Perceived Behavioral Control:** Perceived ease or difficulty of participating in everyday activities
Emotional support from the social environment	Role of family and friends in emotional support	**Subjective Norms:** Influence of family and friends' expectations and their health behavior
		**Perceived Behavioral Control:** Confidence derived from social support in healthcare management
Organizational support from family and friends	Assistance with (long‐term) follow‐up care and appointments	**Subjective Norms:** Family involvement in healthcare decisions
		**Perceived Behavioral Control:** Organizational support enhancing perceived control over health management
Relevance of (long‐term) follow‐up appointments	Importance of regular (long‐term) follow‐up check‐ups	**Attitudes**: Beliefs about the benefits of (long‐term) follow‐up care
		**Perceived Behavioral Control**: Confidence in managing health through regular check‐ups
Relevance of dialog with familiar healthcare professionals	Benefits of discussing concerns with familiar healthcare providers	**Attitudes**: Beliefs about the benefits of transparent communication
		**Perceived Behavioral Control:** Influence of trust and continuity on perceived control over health
Information about (long‐term) follow‐up care	Need for clear, understandable information	**Attitudes:** Beliefs about the importance of age‐adapted individual health communication
		**Perceived Behavioral Control:** Ability to make informed decisions based on perceived information
Shared decision‐making during the transition phase	The desire for participation in transition decisions	**Perceived Behavioral Control:** Empowerment through shared decision‐making
		**Attitudes:** Positive attitudes toward shared decision‐making (e.g., “Being involved makes me feel more in control”)
Challenges of transition	The emotional impact of transitioning to adult care	**Attitudes:** Beliefs about the difficulties of transition
		**Perceived Behavioral Control:** Perceived challenges in adapting to adult care
Exchange with other survivors	Benefits of meeting other survivors	**Subjective Norms:** Influence of peer support on health behaviors
		**Attitudes:** Positive attitudes toward peer interactions

### 3.2 General Survivorship Themes, Relevant to the Survivors During Transition: Relevance of Returning to Everyday Life

Survivors often find it difficult to reintegrate into their social life. Particularly, participation in sports was a recurring theme, representing an area in which respondents experience limitations.

…I would say, especially in the beginning I naturally just wanted the old life back. And it did take some time to get used to the fact that it will never be exactly like before. But yeah, like I said, I found new friends. And by now I think I don't necessarily want to change that much about what now… (Survivor, aged 21, 4 years since diagnosis)

### 3.3 Emotional Support From the Social Environment

Family members often provide emotional support by encouraging and motivating survivors to attend follow‐up appointments. They can also offer a sympathetic ear to discuss fears and concerns.

…And social contacts as well, being with other people a lot, that's definitely—I believe,—something that contributes to health… (Survivor, aged 19, 10.4 years since diagnosis)

### 3.4 Organizational Support From Family and Friends

Family members help to organize appointments or other aspects of care to ensure that (long‐term) follow‐up care organization and related diagnostic and treatment procedures run smoothly. Family members also promote follow‐up care.

…I would say my mother plays a big role [laughs] because she /. Well, my brother has driven me at times, but mostly my mother. I always have to check with school, whether I'm missing a class. But I think if we get the appointment, then we check what we need to organize, and then it will work fine… (Survivor, aged 21, 4 years since diagnosis)

### 3.5 Relevance of (Long‐Term) Follow‐Up Appointments

Some survivors associate health with prevention and control. This includes taking medication regularly, having medical check‐ups, and avoiding risk factors. The interviewees mentioned that regular follow‐up examinations are an important reason for their confidence in their overall health, and to reduce the fear of relapse. (Long‐term) follow‐up appointments offer reassurance that changes in health status will be recognized at an early stage.

…Yes, that is very important. I can't understand many of my friends that I met due to the illness when they for instance say that they don't want to go to follow‐up care. So, I can understand that they are somehow afraid of seeing a bad result and then, I don't know, [coping] simply by suppressing it, saying okay, then there is nothing there. But that doesn't help either. And then I'd rather have confirmation and knowledge that everything is fine than living in uncertainty… (Survivor, aged 20, 6 years since diagnosis)

### 3.6 Relevance of Dialog With Familiar Healthcare Professionals

The majority of survivors mentioned that it helps them to talk to healthcare professionals about their fears and concerns, with a healthcare professional's personality playing an important role. Interviewees who have confidence in their doctors and the quality of medical care were likely to articulate less anxiety. Continuity in medical care is an aspect that is mentioned and contributes to this.

… Because I've known my doctors since I was 13, I think I'm already quite familiar with them. And since then, there were some things here and there, where I had to talk to them anyways. And when I for instance had a bone marrow puncture with one of them, and I experienced minor—well, not minor pain. But it wasn't like I said it's unbearable. And when I noticed during that [procedure] that everything is always taken care of, then I believe in time it turned into a good trusting relationship… (Survivor, aged 19, 5.7 years since diagnosis)

The majority of survivors articulated a need for **information about (long‐term) follow‐up care**.

…Well okay, I would say I don't agree because I wasn't told much, but I also knew a little already and I never asked. So that's a bit /. So somehow, yes, I didn't have any information about what exactly was being done and why. But I never asked about it either, and yes… (Survivor, aged 17, 1.7 years since diagnosis)

However, it was also emphasized that the information should be provided timely and understandably.

…Well, I remember when I was diagnosed, on the very day I was admitted to the hospital, the doctor came and gave me an explanatory talk. And that was very reassuring and very encouraging in the sense that it was going to be positive. It didn't have anything directly to do with follow‐up care, because he also said straight away that it was something chronic that would take a long time if in doubt. And since then I've talked to him more often, also what the plan is now for the coming time. And because we always agreed on how we would do it, I'd say it also really reassuring, because it shows me, okay, I don't have some kind of glorified perspective in saying ‘it'll work out somehow’… (Survivor, aged 19, 5.7 years since diagnosis)

### 3.7 Specific Transition Themes

We also identified several specific transition themes, such as participation in decision‐making during the transition period, interaction with other survivors, and difficulties encountered during the transition process.

### 3.8 Exchange With Other Survivors

Meeting other cancer survivors gives the interviewees confidence. It shows them that a fulfilling life after cancer is possible. Some respondents stated that they would like to receive information about available resources, such as self‐help groups, psychosocial support, and survivorship programs.

… Yes, talking to people about it a bit and if there's anything else, I think it's follow‐up. They also help to have a certain level of security … (Survivor, aged 23, 13.2 years since diagnosis)

### 3.9 Shared Decision‐Making During the Transition Phase

Some interviewees would like to be involved in deciding when the transition begins. They favor shared decision‐making in which the opinions of healthcare providers and survivors are taken into account equally.

…Yes, on the one hand, I can always let the doctors know if I have the feeling that something is up and I know that they will make sure that I get an appointment as quickly as possible so that it can be clarified. But on the other hand, follow‐up care is of course mainly regulated either by guidelines or by letting the doctors decide because they simply know more about the subject than I do. And I can't decide completely on my own. And yes, I would say that I have a certain part in it, but it's not my free decision … (Survivor, aged 19, 5.7 years since diagnosis)

### 3.10 Challenges of Transition

Some survivors perceive the transition as a loss of familiar healthcare providers responsible for them in pediatric wards. This change is mostly described as a considerable challenge as they fear that care provided in adult healthcare facilities will have disadvantages for them.

… Well, the comparison from the adult ward to here, at least here it was like this, to the pediatric ward, was pretty intense. Well, it was much more relaxed and much more personal in the pediatric ward. Sometimes the nurses would stand in the room for 10 or 15 minutes and just talk to you. On the other ward, I don't think I would even have dared to ask for water, something like that. And that's why … (Survivor, aged 45, 17.3 years since diagnosis)

They express concerns about the empathy and time devoted to patients and are anxious that treatment could be less personalized.

… I wouldn't think that's so great. (laughs) Because many people think that in adult oncology, the doctors and nurses don't treat patients so nicely. And I really wouldn't like that. Because then I would probably never go to follow‐up care again … (Survivor, aged 20, 5.3 years since diagnosis)

The majority of survivors had good experiences in pediatric wards and trusted the doctors there. The quality of communication with healthcare providers was seen as crucial. Moreover, they were concerned about having to repeatedly explain the specifics of their illness and treatment to unfamiliar doctors, which could potentially trigger memories of the traumatic aspects of their initial diagnosis and treatment.

… I could imagine that the aspect I just mentioned with familiarity might be a bit more difficult. And, as I just said, this could also make it more difficult to communicate problems. And I could well imagine that it could be stressful to keep repeating how my health is progressing. Because the outpatient clinic knows my case and I'm a familiar face in a way, so I don't have to repeat anything. And I could imagine that this could happen more often in adult oncology. And I already know that from my bone infarctions, going to different doctors again and again is simply very tedious … (Survivor, aged 20, 6.3 years since diagnosis)

The majority of survivors also realized that they have to be proactive in monitoring their health, which could be challenging without the structured support they received in pediatric care.

… Well, you're forced to be a bit more independent because the follow‐up appointments are getting further and further apart. And I think you just have to be more mindful of what's happening to you and then maybe get in touch yourself or get help or something like that … (Survivor, aged 18, 2.6 years since diagnosis)

We also observed a distinction in follow‐up experiences when discussing with survivors the multidisciplinary care provided at specialized facilities within university clinics compared to appointments they must arrange independently elsewhere.

… It's very well organized. So yes, they know exactly how it should be organized, at what intervals, and where I have to go. And even if sometimes I don't always immediately remember to make an appointment myself or if something doesn't fit, I'm always called and there's always some kind of follow‐up. So that's very good … (Survivor, aged 19, 5.7 years since diagnosis)

Survivors expressed a need to be able to integrate follow‐up care into their lives. For instance, they wished to have a comprehensive follow‐up care plan providing an overview of necessary upcoming appointments. A shorter distance to the follow‐up care facilities was mentioned as a facilitator of this process, while costs associated with follow‐up care—such as traveling expenses, overnight stays, or out‐of‐pocket payments for necessary examinations—were considered to be barriers to it.

… So, with stomach pains and not feeling like it sometimes, this constant traveling here and then the examinations, the hours of waiting sometimes, even if you have an appointment, and then have to wait for hours until it's your turn at all. That's also very annoying … (Survivor, aged 20, 6 years since diagnosis)

### 3.11 Motivations to Follow‐Up Based on TBH Dimension

We analyzed motivations to adhere to follow‐up recommendations both qualitatively and quantitatively. Table 3 presents the standardized beta coefficients, whose absolute values provide an estimate of the strength of the relationship between the intention‐to‐attend follow‐up appointments and various potential predictors. Agreement between Likert‐scored answers and the association factor were the strongest predictors of the attitudes toward the transitional process by pediatric cancer survivors (beta = 1.63 resp. –1.50) and the perceived control of the survivors as well (agreement; beta = 1.51).

**Attitudes** refer to the overall positive or negative evaluations of the transition process by pediatric cancer survivors. These attitudes significantly influenced their experiences during the transition. ***Positive attitudes*** toward the transition were fostered by survivors' understanding of the long‐term benefits of follow‐up care. These beliefs motivated them to actively engage in the transition process and adhere to the recommended healthcare routines. Survivors who held positive attitudes toward the transition often considered it as a necessary step for maintaining their health. They recognized the importance of regular (long‐term) follow‐up care in preventing recurrence and managing the late effects of cancer treatment (e.g., “Transitioning to adult care is essential for me to keep track of my health and ensure everything is fine”). *Negative attitudes* were often driven by concerns about losing personalized care and the emotional bonds formed with familiar pediatric healthcare providers. These negative perceptions could lead to resistance or reluctance to engage fully in the transition process. Some survivors expressed negative attitudes toward the transition due to anxiety about leaving familiar pediatric care environments and fear of the unknown in adult healthcare settings (e.g., “Leaving my pediatric doctor feels like losing someone who understands me completely”).

The absolute values of the beta coefficients indicate that the alignment between the level of consent and unconscious stimuli, as well as the related association factors, are strong predictors of the attitudes of pediatric cancer survivors (with beta values of 1.63 and −1.50, respectively).

**Subjective norms** involve the perceived social pressures or expectations from significant others that influence the survivors' behaviors during the transition. ***Family members***, particularly parents, played a crucial role in encouraging and supporting survivors through the transition. The strong influence of family support provided survivors with a sense of obligation and motivation to adhere to (long‐term) follow‐up care schedules. This external validation and encouragement were pivotal in shaping positive transition experiences (e.g., “Knowing my parents want the best for me makes me more committed to attending follow‐ups”), while at the same time, it does not strengthen personal responsibility for the life‐long management of late effects. Interactions with ***peers*** who had undergone similar transitions provided emotional support and practical advice. Positive peer influences and the shared experiences of other survivors helped normalize the transition process, reducing anxiety and promoting a more proactive approach to (long‐term) follow‐up care (e.g., “Hearing from others about their positive experiences makes me hopeful about my transition”).

Subjective norms tend to be higher with positive associations to relevant terms of cancer and with increasing age at diagnosis.

**Perceived behavioral control** refers to the survivors' perception of their ability to manage the transition and adhere to (long‐term) follow‐up care, influenced by internal and external factors. ***High perceived control*** was often associated with survivors' confidence in their ability to manage their health independently, supported by organized healthcare systems and family assistance (e.g., “Having a structured plan from my healthcare team makes the transition process smoother”). This control enhanced their engagement and compliance with (long‐term) follow‐up care. Survivors who felt confident in their organizational skills and had strong social support systems perceived the transition as manageable. ***Low perceived control*** was linked to the presence of significant barriers that hindered survivors' ability to engage effectively in the transition. These barriers necessitated additional support and resources to enhance their control and facilitate a smoother transition. Survivors who faced logistical, financial, or emotional barriers perceived the transition as challenging and overwhelming (e.g., “Managing appointments along with my studies and work is tough”).

According to the standardized beta coefficients, perceived behavioral control increases with higher agreement between the extent of consent and unconscious stimuli of cancer survivors.

## 4 Discussion

In this article, we focused on analyses of 10 from 38 interviews conducted with pediatric cancer survivors aged 17–45 during their transition from childhood to adult care facilities, aiming to explore their needs in follow‐up care during this crucial period and to understand factors affecting their motivations to adhere to (long‐term) follow‐up recommendations. A mixed‐method approach to the transcript analyses allowed us to gain a deep understanding of survivors' experiences with pediatric follow‐up care and behavioral factors affecting their adherence during the transitional phase.

### 4.1 Needs of Transition Survivors

Most of the survivors interviewed mentioned the importance of returning to everyday life and the supportive role of family in facilitating this process. These results alienate with a similar study on the pediatric cancer survivor population in Switzerland [32, 33] the interviewees emphasized the difficulty in reintegrating into social life and the crucial role in this process of emotional and organizational support from the immediate social environment. Moreover, survivors in our study noted that meeting other cancer survivors positively impacted their confidence. This supports the findings of Zebrack et al. [34] which indicate that support groups and survivor networks significantly enhance mental health and attendance at follow‐up appointments. Interestingly, survivors who participated in our study mostly recognized the importance of follow‐up appointments for health maintenance and as a source of support in returning to “normal activities,” while results of previous research on the transition group of pediatric cancer survivors showed other trends: Many survivors perceive themselves as entirely healthy or invincible, failing to acknowledge their risk of significant long‐term health issues and not following the recommended cancer‐related follow‐up care [7, 35]. This may be explained by the age of our cohort diagnosed and treated in recent times when the relevance of long‐term follow‐up was more and more included in health communication/education during acute cancer treatment.

Our findings align with previous research, which demonstrated that psychological, emotional, and relational interactions between patients and healthcare providers are very important for pediatric cancer survivors [36, 37]. However, the influence that these interactions have on transition experiences is twofold. On the one hand, pediatric healthcare providers are often perceived as akin to family members, forming significant emotional bonds with patients [38], thus improving adherence to care in pediatric wards. At the same time, strong relationships with pediatric healthcare providers caused survivors to experience anxiety before transitioning [39]. Participants in our study attributed this anxiety to trust issues and the need to repeatedly explain their medical history to unfamiliar healthcare professionals, which may stimulate traumatic memories. Furthermore, survivors felt that when adult healthcare providers repeatedly asked the same questions about their medical history or managing their condition during appointments, it suggested to them that these providers were unfamiliar with their case and appeared less competent compared to their pediatric healthcare providers [39]. Addressing the emotional aspects linked to a loss of familiarity with daily medical routines within adult‐oriented healthcare systems is challenging. Nevertheless, emphasizing positive peer communication and consistently integrating (digital‐supported) measures that encourage such communication and exchange could potentially pave the way for future interventions aimed at enhancing transitional processes.

Participants in our study expressed a need for more information from healthcare providers about their past disease, possible late effects, and available psychosocial support like self‐help groups or psychotherapy. In the study by Svedberg et al. involving 213 young adult survivors of pediatric cancer, it was found that the survivors would have appreciated more follow‐up information tailored to their specific needs and psychosocial health [40]. They also emphasized the need for better education on how follow‐up appointments are organized and structured. Furthermore, previous research has shown that pediatric cancer survivors aged 17–20 years possess significantly greater knowledge and interest in the transition process, along with enhanced self‐management skills for scheduling their appointments [38]. Survivors interviewed in our study acknowledged this as a crucial requirement for proactively monitoring their condition. To do so, the need for timely information on the transition process was articulated. Similarly, previous research has shown that understanding the differences between pediatric and adult care could ease the transition [41, 42].

Participants in our study who were interviewed in the university hospitals with established long‐term follow‐up facilities underlined the face‐to‐face presence during transition appointments of both pediatric and adult providers. It ensured them that information about their condition would be effectively communicated. Additionally, it facilitated the establishment of relationships with their new healthcare providers [2, 43]. However, as previously discussed, not all challenging emotional aspects appear to be addressed by this transitional process.

Upon transitioning to the adult healthcare setting, survivors acknowledged a shift in the interaction and communication style of adult healthcare providers compared to their pediatric colleagues. They believed that pediatric healthcare providers could help ease this transition by involving them more actively at an earlier stage [44]. At the same time, survivors acknowledged the importance of the care provided by their parents and pediatric healthcare providers but reported that this has left them less prepared for autonomy in adult life. Consequently, parents continue to play a significant role in communication with healthcare services even as the survivors reach adulthood. Previous research has shown that parents typically overtake the functions of advocating for survivors in adult healthcare settings, aiding in remembering their medical history, and preventing misunderstandings with healthcare providers [2, 43, 45]. At the same time, survivors acknowledged the shift in their role and recognized the significance of embracing independence. They viewed this transition as a natural progression and understood that developing self‐management skills was a part of growing. Survivors perceived this change in roles as a gradual process and valued acquiring knowledge and skills to enhance their ability to self‐manage their condition and interact effectively with their new healthcare providers [41, 46]. However, challenges arise when parents significantly limit their responsiveness in guiding their children, and they struggle to recognize the appropriate point to reduce this support [47].

### 4.2 Motivations to Adhere to (Long‐Term) Follow‐Up Recommendations

The TBH has previously demonstrated its utility in understanding the factors that influence follow‐up attendance among pediatric cancer survivors [32, 33]. According to our research in this subgroup, we extracted the following factors affecting adherence to (long‐term) follow‐up recommendations:

- Personal evaluations of the positive and negative consequences of follow‐up care. Similar to previous research [32], a positive attitude toward follow‐up care was found to increase the intention to attend. We also found that the more positive the unconscious prime is, the better the actual attitude toward follow‐up and the stronger the intention to adhere to follow‐up recommendations. However, the relatively small sample of survivors interviewed limits the robustness of the conclusions resulting from the quantitative analyses.
- Perceived social pressure from important others (e.g., parents and family) to attend follow‐up care. This includes the belief that significant others expect them to attend as well as the health attitudes of “important” others toward their health, which adds to the results of a similar study conducted on a Swiss cohort of pediatric cancer survivors [48]. We have also found that the longer survivors are from their initial cancer diagnosis, the more significant this factor becomes in their motivation to adhere to (long‐term) follow‐up recommendations.
- Ability and confidence in attending follow‐up care were key factors resulting from the behavioral control dimension. Behavioral control involves both the actual control over the behavior and the confidence to perform it. Similar to other studies [32], higher perceived control was linked to higher intention and actual attendance. Based on our results, it is perceived higher if the unconscious primes fit to align with actual behavior.

The findings from this study highlight several implications for clinical practice in supporting pediatric cancer survivors during their transition to adult healthcare. There is a clear need to enhance the organization of transitional systems to ensure that survivors receive comprehensive support during this critical shift. Further dissemination of structured practices (e.g., multidisciplinary (long‐term) follow‐up centers can facilitate better communication and coordination among healthcare providers). This aligns with the findings of a review by Ehrhardt et al. [49], which emphasizes the need for systematic, age‐appropriate transitional practices that utilize well‐established clinical care models, survivorship care plans, and guidelines to ensure effective transitions between providers. Additionally, equipping family members with the necessary tools and resources will empower them to effectively support survivors throughout this process. Emphasizing the importance of patient support groups, particularly during the transition period, can provide invaluable emotional support and practical guidance for survivors. Furthermore, actively involving patients in the decision‐making process related to their care and transition initiation fosters a sense of autonomy and encourages proactive engagement in their healthcare journey. Addressing these areas in clinical practice will not only improve the transition experience for pediatric cancer survivors but also enhance their long‐term health outcomes and overall quality of life.

### 4.3 Strengths and Limitations

The primary strength of this study lies in the structured interview guidelines that incorporate Likert‐scale questions. These guidelines enable a combined qualitative and quantitative analysis approach while mitigating potential biases related to social desirability. Additionally, the integration of multiple interconnected theories provides a comprehensive understanding of the factors influencing adherence among pediatric cancer survivors. Another strength is the involvement of a multidisciplinary team of researchers, including experts from healthcare research, medical professionals, and mental health experts both involved in care and research, which enriches the study's depth and breadth.

We are also aware of several limitations. Firstly, there is a potential limitation regarding participants feeling constrained to share negative information, possibly leading to socially acceptable responses. To address this concern, the interview guidelines were designed with various types of questions, including free associations and Likert‐scale questions. Secondly, the interviews were conducted on a volunteer basis and primarily in hospitals with established long‐term follow‐up care structures. As a result, the perspectives gathered may not fully represent pediatric cancer survivors who are unaware of the late effects of cancer in childhood and adolescence, have no knowledge of, or are not attending (long‐term) follow‐up care. This situation could also influence responses, as survivors may fear damaging their relationships with follow‐up healthcare providers by providing “undesirable” answers. Apart from this, survivors may not have been ready to fully disclose potential traumatic experiences of cancer or may still face stigma associated with cancer. Additionally, important factors such as race and economic status, which may shape participants' feelings toward the healthcare system and/or access to certain healthcare services, were not discussed, potentially overlooking significant influences on survivors' experiences. Furthermore, due to recruitment constraints in the analyzed age group, no survivors of pediatric CNS tumors, who may experience a significant cumulative burden of late effects and impairments, were included, despite their potentially distinct motivational circumstances. Lastly, the small sample size (*n* = 10) limits the generalizability of the findings. Because of this limited sample size as well as selective (non)participation of nonadherent survivors, the results from both qualitative and quantitative analyses should be interpreted as general trends rather than definitive conclusions. Being a part of a larger VersKiK project, these results provided us a framework for working on suggestions for further improvement of organization in transition care after pediatric cancer. Additionally, qualitative insight may provide valuable information for transition patients suffering from other diseases with life‐long care demands.

## 5 Conclusion

The integration of “primes,” attitudes, subjective norms, and perceived behavioral control provides a comprehensive understanding of the factors influencing transition experiences after pediatric cancer. Positive attitudes and strong subjective norms from family and peers, coupled with high perceived behavioral control, seem to contribute to smoother transitions and better adherence to follow‐up care. Conversely, negative attitudes, lack of social support, and low perceived control posed significant challenges, highlighting the need for targeted interventions to address these issues. By addressing these factors, healthcare providers can develop more effective strategies to support pediatric cancer survivors in their journey from childhood to adult healthcare facilities, ultimately improving their long‐term health outcomes and quality of life.

## Author Contributions

**Ekaterina Aleshchenko:** conceptualization (lead), data curation (lead), funding acquisition (supporting), methodology (lead), project administration (lead), resources (lead), writing – original draft (lead). **Thorsten Langer:** conceptualization (supporting), resources (supporting), supervision (supporting), writing – review and editing (supporting). **Gabriele Calaminus:** conceptualization (supporting), supervision (equal), writing – review and editing (supporting). **Juliane Glogner:** resources (supporting), writing – review and editing (supporting). **Henrike Haugke:** data curation (supporting), writing – review and editing (supporting). **Pietro Trocchi:** data curation (supporting), writing – original draft (supporting), writing – review and editing (supporting). **Enno Swart:** conceptualization (supporting), funding acquisition (lead), project administration (equal), supervision (lead), validation (supporting), writing – review and editing (supporting). **Katja Baust:** conceptualization (supporting), data curation (supporting), methodology (supporting), resources (supporting), writing – original draft (supporting), writing – review and editing (lead).

## Ethics Statement

The VersKiK study was approved by the Ethics Committee Otto von Guericke University on 7.2.2021 (103/21), the Ethics Committee of Johannes Gutenberg University Mainz on 6.16.2021 (2021–16,035), the Ethics Committee University of Lübeck on 11.10.2021 (21–451), and the Ethics Committee University of Hospital Bonn on 2.28.2022 (05/22). For each part of the qualitative study, a separate written informed consent is prepared and approved accordingly by the Ethics Committees named above.

## Conflicts of Interest

The authors declare no conflicts of interest.

## Data Availability

The anonymised datasets analysed during the current study are available from the corresponding author on reasonable request.
